# Adding MYC/BCL2 double expression to NCCN-IPI may not improve prognostic value to an acceptable level

**DOI:** 10.1007/s44313-024-00006-w

**Published:** 2024-02-19

**Authors:** Naree Warnnissorn, Nonglak Kanitsap, Pimjai Niparuck, Paisarn Boonsakan, Prapasri Kulalert, Wasithep Limvorapitak, Lantarima Bhoopat, Supawee Saengboon, Chinnawut Suriyonplengsaeng, Pichika Chantrathammachart, Teeraya Puavilai, Suporn Chuncharunee

**Affiliations:** 1 Department of Pathology, Faculty of Medicine, Thammasat University, Pathumthani, Thailand; 2 Division of Hematology, Department of Medicine, Faculty of Medicine, Thammasat University, Pathumthani, Thailand; 3 Division of Hematology, Department of Medicine, Ramathibodi Hospital, Mahidol University, Bangkok, Thailand; 4 Department of Pathology, Ramathibodi Hospital, Mahidol University, Bangkok, Thailand; 5 Department of Clinical Epidemiology, Faculty of Medicine, Thammasat University, Pathumthani, Thailand; 6 Department of Anatomy, Faculty of Science, Mahidol University, Bangkok, Thailand

**Keywords:** DLBCL, MYC/BCL2 double expression, R-CHOP, Prognosis, NCCN-IPI, REMARK

## Abstract

**Background:**

MYC/BCL2 double expression (DE) is associated with poor prognosis in patients with diffuse large B-cell lymphoma (DLBCL) receiving rituximab, cyclophosphamide, doxorubicin, vincristine, and prednisolone (R-CHOP). This study aimed to determine whether the addition of DE to the National Comprehensive Cancer Network Internal Prognostic Index (NCCN-IPI) could improve the prediction of disease progression in patients with DLBCL treated with R-CHOP.

**Methods:**

This confirmatory prognostic factor study retrospectively recruited patients with newly diagnosed DLBCL between January 1, 2014, and January 31, 2018, at Ramathibodi Hospital (RA) and Thammasat University Hospital (TU). The follow-up period ended on July 1, 2022. Tumors expressing MYC ≥ 40% and BCL2 ≥ 50% were classified as DE. We calculated the hazard ratios (HR) for progression-free survival (PFS) from the date of diagnosis to refractory disease, relapse, or death. Discrimination of the 5-year prediction was based on Cox models using Harrell’s concordance index (c-index).

**Results:**

A total of 111 patients had DE (39%), NCCN-IPI (8%), and disease progression (46%). The NCCN-IPI adjusted HR of DE was 1.6 (95% confidence interval [CI]: 0.9–2.8; *P* = 0.117). The baseline NCCN-IPI c-index was 0.63. Adding DE to the NCCN-IPI slightly increased Harrell’s concordance index (c-index) to 0.66 (*P* = 0.119).

**Conclusions:**

Adding DE to the NCCN-IPI may not improve the prognostic value to an acceptable level in resource-limited settings. Multiple independent confirmatory studies from a large cohort of lymphoma registries have provided additional evidence for the clinical utility of DE.

**Supplementary Information:**

The online version contains supplementary material available at 10.1007/s44313-024-00006-w.

## Introduction

Diffuse large B-cell lymphoma (DLBCL) is molecularly heterogeneous. The patients exhibit variable clinicopathological features and treatment outcomes. Disease progression occurs in approximately one-third to half of patients treated with rituximab, cyclophosphamide, doxorubicin, vincristine, and prednisolone (R-CHOP) [1]. Risk stratification according to the National Comprehensive Cancer Network internal prognostic index (NCCN-IPI), is based on age, stage, lactate dehydrogenase (LDH), extranodal sites, and Eastern Cooperative Oncology Group Performance Status (ECOG) [2]. However, the discrimination of NCCN-IPI was below the acceptable level (c-index less than 0.7) [3, 4]. Adding marker(s) to the NCCN-IPI may help update the model to select high-risk patients for alternative therapies.

Among the IHC markers, the double expression of MYC and BCL2 (double expression [DE]) has been reported to result in lower overall survival (OS) in the rituximab era [5]. The poor prognostic effects of DE may be due to the function of MYC in driving cell proliferation and BCL2 as an anti-apoptotic protein. DE was detected in 31% of DLBCL [6] and 70% of high-grade B-cell lymphomas with *MYC* and *BCL2* and/or *BCL6* rearrangements (so-called double-hit lymphoma [DHL] and triple-hit lymphoma [THL]) [7]. DHL/THL accounts for 7.9% of DLBCL [7] and has an aggressive clinical course [8]. Recent studies excluding DHL/THL found no significant association between DE and OS or progression-free survival (PFS) [9, 10]. Fluorescence in situ hybridization (FISH) of *MYC* and *BCL2* and/or *BCL6* rearrangements is a diagnostic requirement for DHL/THL. However, it could not be tested in all patients in our clinical setting because of its high cost and limited availability in routine diagnostic laboratories.

This study aimed to determine if adding DE to the NCCN-IPI could improve the prediction of progression in patients with DLBCL treated with R-CHOP in resource-limited settings.

## Methods

### Study design and setting

This study retrospectively recruited consecutive patients diagnosed with de novo DLBCL, not otherwise specified, between January 1, 2014, and January 31, 2018, from Thammasat University Hospital (TU) and Ramathibodi Hospital (RA). The diagnosis was based on the World Health Organization (WHO) classification [11]. The follow-up period ended on July 1, 2022.

We included patients at least 18 years old who received R-CHOP as first-line therapy and whose MYC/BCL2 results were available. The number of R-CHOP cycles and additional treatments were determined according to stage, tumor size, and outcome as the standard of care. Additional treatments include radiation therapy (RT), salvage regimens, surgical resection, and hematopoietic stem cell transplantation (HCT).

### Data collection

Diagnosis, age, LDH level, stage, extranodal involvement, performance status, DE, treatment, follow-up physical examination, laboratory, and radiologic findings) from electronic medical records. Physical examination, bone marrow (BM) biopsy, and computed tomography (CT) or fluorodeoxyglucose (FDG) positron emission tomography (PET)-CT assessed stage and response. The treatment responses were according to the Lugano criteria: complete remission (CR), partial response (PR), stable disease, and progressive disease (PD) [12]. FISH and PET-CT were not available for most patients because of their high costs and limited indications for reimbursement. PFS was defined as the period from diagnosis to the first occurrence of disease progression (relapsed or refractory disease) or death from any cause.

### Immunohistochemistry

Immunohistochemistry (IHC) staining, as part of routine diagnostics, was performed on freshly cut 4-μm thick formalin-fixed paraffin-embedded (FFPE) whole tissue sections. The primary antibodies used were anti-c-MYC (clone Y69, 1:100, Biocare) and anti-BCL2 (clone 124, 1:100, Dako). IHC interpretation was based on two high-power fields (400x) of viable tumor cells expressing moderate or strong intensity. MYC was positive when nuclear staining was ≥ 40%, while BCL2 was positive when cytoplasmic staining was ≥ 50% [13]. The expression of MYC/BCL2 was binary for DE (MYC + /BCL2 +) and non-DE (other than MYC + /BCL2 +). Hematopathologists at TU (N.W.) and RA (P.B.) interpreted the IHC results independently and blindly for clinical outcomes.

### Statistical analysis

We measured the association between DE and other variables using Fisher’s exact test for binary or categorical data, and the Wilcoxon rank-sum test for continuous data. Survival analysis and Kaplan–Meier (KM) curves were compared using a nonparametric log-rank test. The 5-year (5-y) PFS life table was reported as percentages with a 95% confidence interval (CI) and log-rank test *P*. Differences were considered statistically significant at *P* < 0.05. Unadjusted and adjusted effects were estimated using the Cox proportional hazards model to report hazard rations (HRs) with 95% CI and *P*. We adjusted the DE using the NCCN-IPI (categorical). We assessed the discrimination of NCCN-IPI with DE and NCCN-IPI Cox models for 5-y PFS using Harrell’s concordance index (c-index) [14]. Discrimination refers to how well the predictions discriminate (separate) between participants who do and do not develop progression (the outcome of interest) [15]. The c-index of 1 is perfect, 0.7 is acceptable, 0.6 is poor, and 0.5 is no better than a coin flip. The c-index difference between the two models was calculated using a linear combination [16]. All the patients had DE and NCCN-IPI results (complete case analysis). This study followed the Reporting Recommendations for Tumor Marker Prognostic Studies (REMARK) and reported an overview of patients, markers (M), further variables (v), initial data analysis (IDA), and analysis (A) in the REMARK profile [17] (Supplementary Materials).

### Sample size estimation

Assuming a progression probability of 40% to detect an HR of 2.0, a standard deviation of 0.7, power of 80%, and an alpha error of 5%, the estimated events would be at least 34 progressions from a total number of 84 patients.

## Results

### Participants

Of the 319 patients diagnosed with de novo DLBCL, we excluded 208 because 133 patients did not receive R-CHOP (65 received CHOP; 4 dose-adjusted etoposide, prednisone, vincristine, cyclophosphamide, and doxorubicin; 20 palliative; 44 no treatment [27 deaths, 9 lost to follow-up, and 8 transferred to other hospitals]), and 75 cases had no MCY/BCL2 results. The main reasons for not administering rituximab were its high cost and lack of access. Patients treated with CHOP were mostly diagnosed before the Thailand Universal Health Care Coverage Scheme provided rituximab to all patients with DLBCL in 2017.

**Fig. 1:**
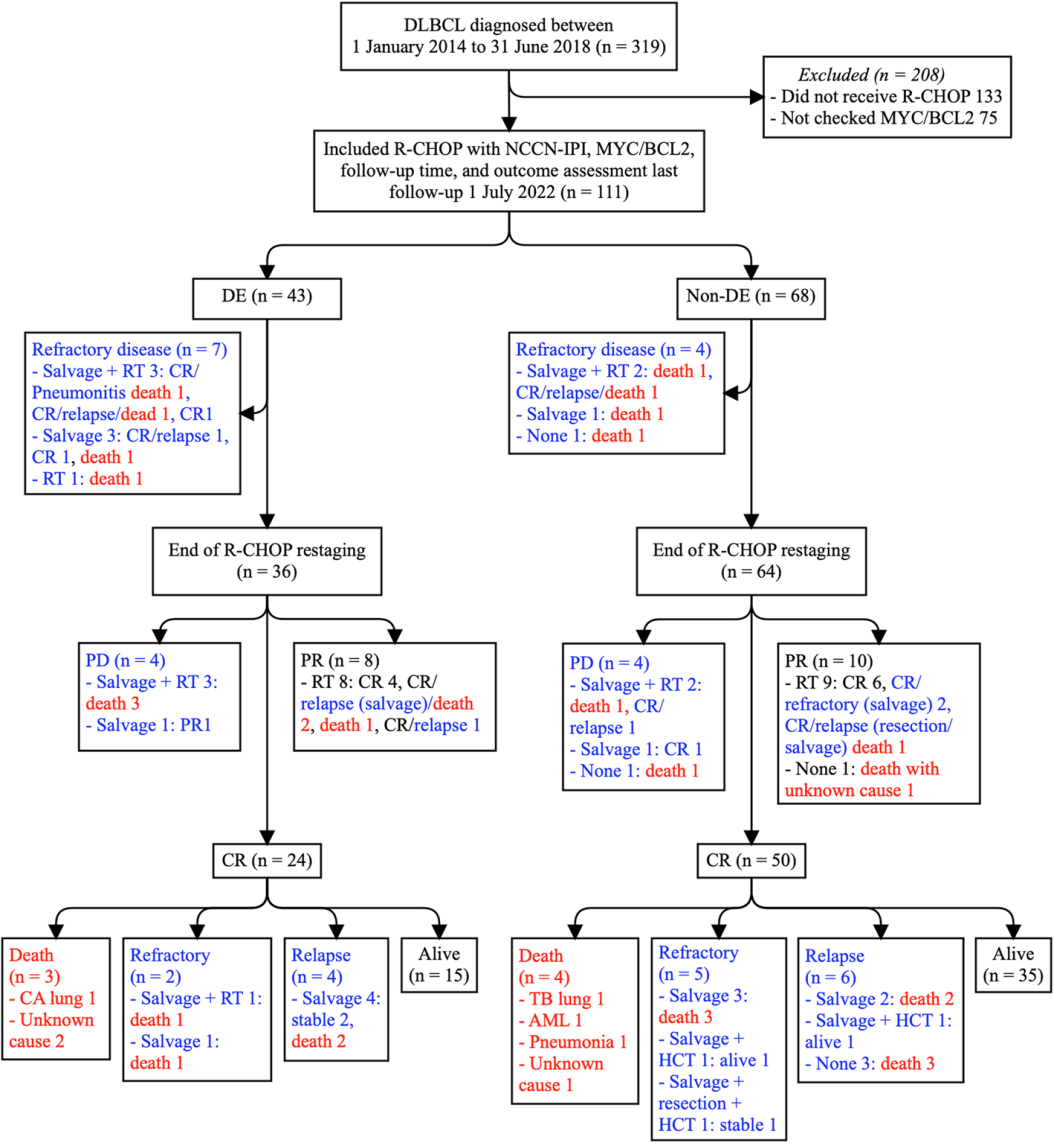
Participant flow diagram

The final cohort included 111 patients with DEs (*n* = 43) and 68 patients without DE (Fig. 1 and Table 1). The median follow-up time of the entire cohort was 4.9 years (range, 0.4 to 8.2), and for those without progression, it was 5.3 years (range, 2.6–7.6). Most patients (87%) received treatment at Ramathibodi Hospital. Primary refractory disease occurred before the end of R-CHOP treatment in 11 patients (seven DEs and four non-DEs). Most patients with refractory disease or relapse received salvage regimens as an additional treatment. Deaths were related to lymphoma in 29 patients (14 DEs and 15 non-DEs) and from other causes in 8 patients (3 DEs in CR followed by death: 1 lung cancer [CA] and 2 with unknown cause; 1 non-DE in PR followed by death with unknown cause, and 4 non-DE in CR followed by death: 1 tuberculosis (TB) of the lung, 1 acute myeloid leukemia (AML), 1 pneumonia, and 1 unknown cause) (Fig. 1).

**Table 1:** Patient characteristics

Characteristic	All Number (%)	DE Number (%)	Non-DE Number (%)	*P*
Total	111 (100)	43 (39)	68 (61)	NA
Sex				0.437
Male	51 (46)	22 (51)	29 (43)	
Female	60 (54)	21 (49)	39 (57)	
Age, median (range)	62 (26–88)	66 (28–80)	62 (26–88)	0.374
LDH ratio > 1	67 (60)	25 (58)	42 (62)	0.842
Stage III–IV	60 (54)	21 (49)	39 (57)	0.437
Extranodal site	45 (41)	13 (30)	32 (47)	0.112
ECOG ≥ 2	13 (12)	6 (14)	7 (10)	0.561
NCCN-IPI low	3 (3)	1 (1)	2 (3)	0.721
LI	60 (54)	21 (49)	39 (57)	
HI	39 (35)	18 (42)	21 (31)	
High	9 (8)	3 (7)	6 (9)	
Tumor size ≥ 10 cm	28 (25)	8 (19)	20 (29)	0.263
Follow-up without progression, median (range)	5.3 (2.6–7.6)	5.1 (3.0–6.6)	5.7 (2.6–7.6)	0.076
Complete response	97 (87)	35 (81)	62 (91)	0.151
Progression	51 (46)	24 (56)	27 (40)	0.119
Death	37 (33)	17 (40)	20 (29)	0.305

*DE* double expression of MYC and BCL2 proteins, *ECOG* Eastern Cooperative Oncology Group Performance Status, *HI* High intermediate, *LDH* Lactate dehydrogenase, *LI* Low intermediate, *NA* not applicable, *NCCN-IPI* National Comprehensive Cancer Network International Prognostic Index

The cohort comprised 51 men and 60 women, with a median age of 62 years (range, 26–88 years). Most patients were older than 60 years of age (59%) and had elevated LDH (60%). The majority of patients were in stages III–IV (54%). According to the NCCN-IPI, the patients were stratified into high-risk (8%), high-intermediate-risk (HI) (35%), low-intermediate-risk (LI) (54%), and low-risk (3%) groups. Approximately a quarter had bulky disease (tumor size ≥ 10 cm). FISH for *MYC/BCL2/BCL6* gene rearrangements was available for 21 patients (19%) with rearranged *BCL6* in two DE cases, no rearrangement in 14 DEs and two non-DE cases, and unsatisfactory results in three DE cases. PET-CT was available at staging in one patient (1%) and at follow-up or restaging in 26 patients (24%). Treatment outcomes were CR in 97 patients (87%), disease progression in 51 (46%), and death in 37 (33%) (Table 1). Among the 60 patients without progression, 50 achieved CR and remained alive, and 10 patients had PR followed by RT and then CR. The estimated 5-y PFS was 47% (95% CI, 31–61%), and 5-y OS was 61% (95% CI, 44–74%).

DE had no significant relationship with clinical prognostic variables (age, LDH, stage, extranodal site, and ECOG performance status) or the NCCN-IPI. The outcomes of the patients with DE were slightly poorer than those of the patients without DE (Table 1).

### Survival analysis

**Fig. 2:**
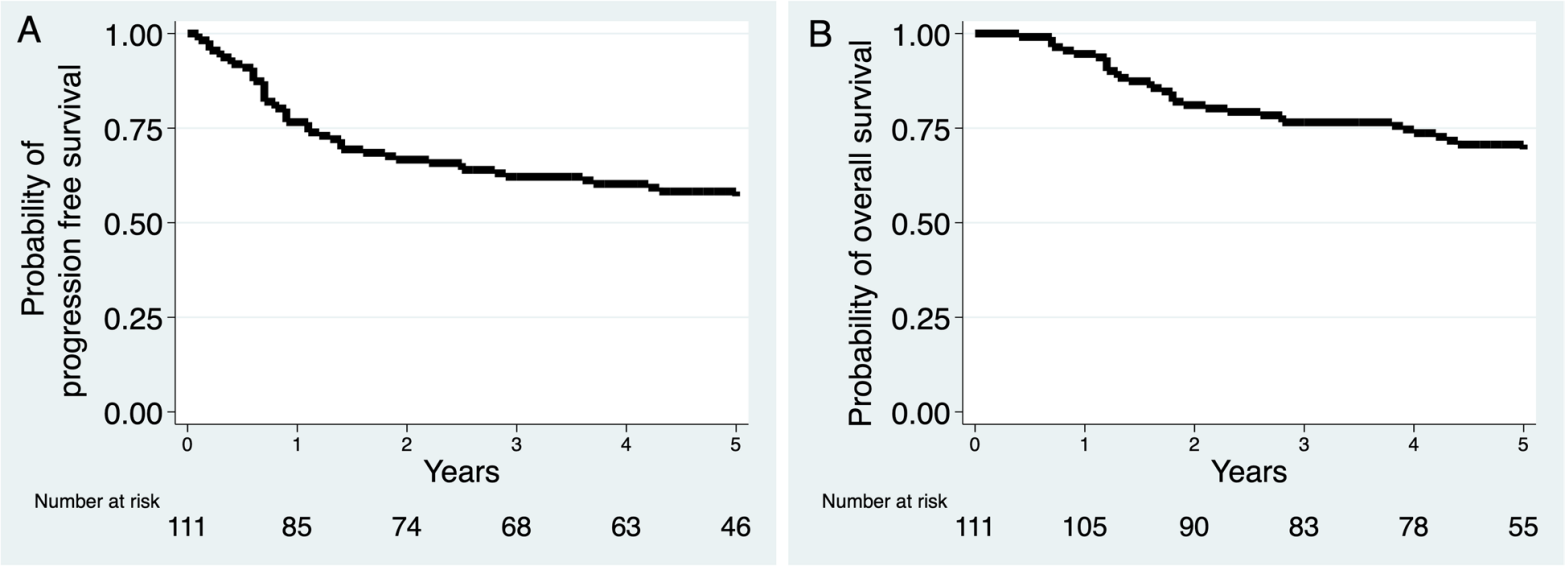
Kaplan–Meier survival curves: **A** progression-free survival and **B** overall survival

The PFS and OS curves of the entire cohort rapidly declined within two years after diagnosis before leveling out and did not reach the median PFS and OS (Fig. 2). The estimated 5-y probability of PFS was 55% (95% CI, 44–64%) and the 5-y OS was 68% (95% CI, 58–76%).

**Fig. 3:**
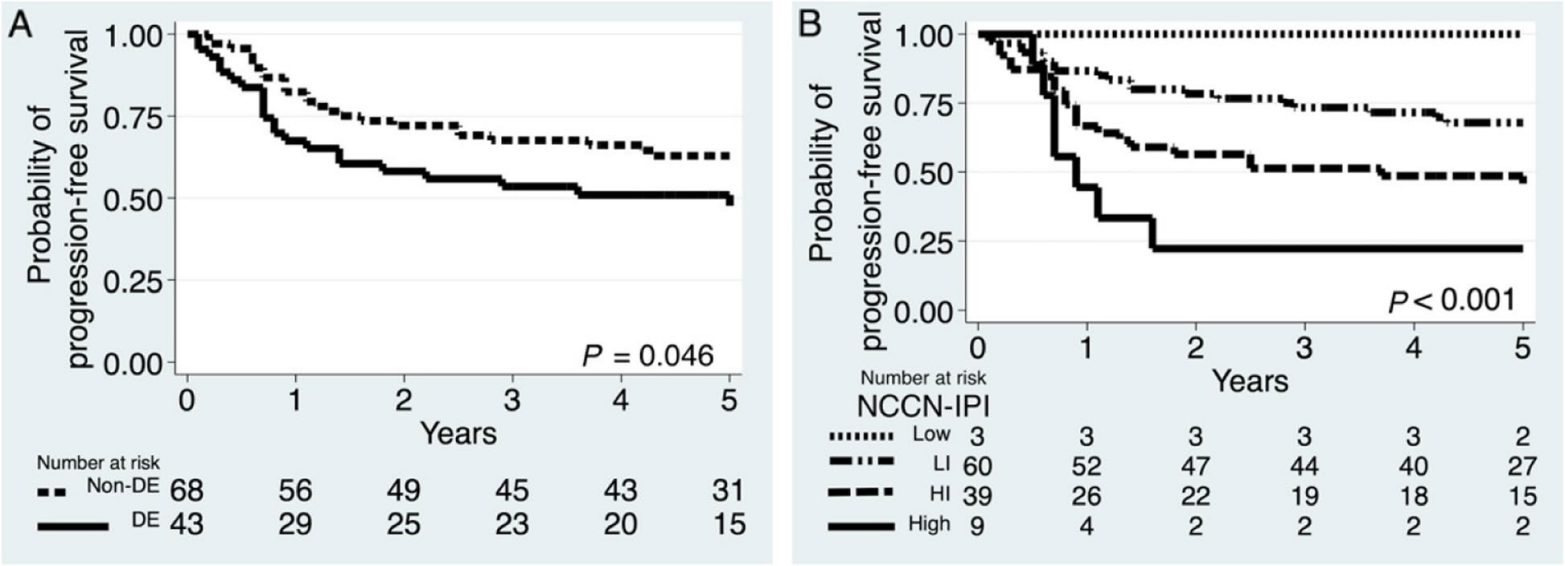
Kaplan–Meier progression-free survival curves: **A** DE (*P* = 0.046) and **B** NCCN-IPI (*P* < 0.001)

The PFS curves and 5-y-PFS probabilities were marginally different according to DE (*P* = 0.046) and significantly different according to NCCN-IPI (*P* < 0.001) (Fig. 3 and Table 2).

**Table 2:** Estimated 5-year progression-free survival probabilities of DE and NCCN-IPI

Variables	Number (%)	5-y PFS (95% CI)	*P*
Non-DE	43 (39)	60 (46–71)	0.046
DE	68 (61)	47 (31–62)	
NCCN-IPI Low	3 (3)	100 (.-.)	< 0.001
LI	60 (54)	64 (50–75)	
HI	39 (35)	46 (30–61)	
High	9 (8)	22 (3–51)	

*DE* double expression of MYC and BCL2 proteins, *HI* high-intermediate, *LI* low-intermediate, *NCCN-IPI* National Comprehensive Cancer Network Internal Prognostic Index, *PFS* Progression-free survival

### Prognostic strength

In an unadjusted analysis of DE for PFS, HR was 1.7 (95% CI, 1.0–3.0; *P* = 0.051), and the discrimination c-index was 0.56 (95% CI, 0.49–0.64). The NCCN-IPI adjusted HR of DE was 1.6 (95% CI, 0.9–2.8; *P* = 0.117). The baseline NCCN-IPI c-index was 0.63 (95% CI, 0.56–0.71), while adding DE to the NCCN-IPI (NCCN-IPI + DE) c-index slightly increased it to 0.66 (95% CI, 0.58–0.74; *P* = 0.119) (Table 3). The univariable HR of NCCN-IPI for PFS was 2.3 (95% CI, 1.5 to 3.2; *P* < 0.001).

**Table 3:** Prognostic strength of DE for prediction of progression-free survival

Effect	Progression-free survival	
	Value (95% CI)	*P*
Unadjusted		
HR of DE	1.7 (1.0–3.0)	0.051
c-index of DE	0.56 (0.49–0.64)	NA
Adjusted		
NCCN-IPI adjusted HR of DE	1.6 (0.9–2.8)	0.117
c-index of NCCN-IPI	0.63 (0.56–0.71)	NA
c-index of NCCN-IPI + DE	0.66 (0.58–0.74)	NA
c-index difference	0.03 (-0.01–0.06)	0.119

*c-index* concordance index, *DE* double expression of MYC and BCL2 proteins, *HR* hazard ratio, *NA* not applicable, *NCCN-IPI* National Comprehensive Cancer Network Internal Prognostic Index

## Discussion

Double expression of MYC and BCL2 as promising prognostic markers from previous exploratory studies and meta-analyses requires further confirmatory studies to prove their clinical utility. This study evaluated whether adding DE to the NCCN-IPI in patients with DLBCL treated with R-CHOP could improve prognostic prediction to an acceptable level. Our cohort of 111 patients had a high proportion of DE (39%), with a high percentage of NCCN-IPI (8%) and HI (35%) and progression in 46%. The NCCN-IPI adjusted HR of DE on progression-free survival was 1.6 (95% CI, 0.9–2.8; *P* = 0.117). Adding DE to NCCN-IPI slightly increased the discrimination (c-index baseline NCCN-IPI 0.63 to NCCN-IPI + DE 0.66, *P* = 0.119), which remained below an acceptable level (c-index ≥ 0.70). Thus, adding DE to the NCCN-IPI did not increase the prognostic strength in predicting progression.

The meta-analysis reported a significant association of DE with poor OS (pooled HR 2.58; 95% CI, 2.19–3.04; I^2^ 17.2%; *P* = 0.275) [5]. The analysis was based on exploratory studies that recruited patients diagnosed between 1998 and 2009, which may have included patients with DHL/THL in the population. The clinical outcome of DHL/THL was extremely poor compared to that of DE and non-DE patients with DLBCL (5-y OS and PFS rates: DHL/THL 27% and 18% vs. DE 36% and 32% vs. non-DE 71% and 65%, respectively) [13]. The WHO classification recognized DHL/THL as a provisional entity in 2017 because of its distinct biology with a very aggressive clinical course and it should not be classified as DLBCL [18]. DHL/THL represents 7.9% of DLBCL cases and has a DE of 70% [7].

FISH for *MYC* and *BCL2* and/or *BCL6* rearrangements was not performed in all DLBCL cases because of the high cost and low laboratory availability. Affordable ubiquitous IHC staining for MYC/BCL2 and the Hans algorithm are screening tools for selecting those with DE and germinal center B-cell type results for FISH testing [7]. The limitations of IHC include variations in tissue fixation duration and subjective interpretation.

Recent studies that separately investigated DHL/THL found no significant association between DE and inferior survival [9, 10]. The presence of DHL/THL in the study population may have contributed to the poor outcomes in patients with DE. In addition, these previous studies performed IHC on tissue microarrays and used different MYC/BCL2 cut-offs (40%/70% [19], 50%/30% [20], and 70%/70% [21]). The second meta-analysis indirectly reaffirmed the predictive power of DE, as non-DE had a higher probability of CR (odds ratio 2.7; 95% CI, 1.6–4.7) with significant heterogeneity between eight studies (I^2^ = 68%, *P* < 0.01), but did not report an adverse prognostic effect in association with PFS or OS [6].

A previous study on the addition of double expressor lymphoma (DEL) score (no DE: 0, either MYC ≥ 40% or BCL2 ≥ 70%: 1, and DE: 2) to the NCCN-IPI was conducted in a cohort of 277 patients with DE (22%), NCCN-IPI high risk (13%), and 5-y PFS (55%). They showed a strong association between DE (HR adjusted for the NCCN-IPI: 2.4, *P* = 0.007) and a significant improvement in discrimination toward the 5-y PFS; c-index NCCN-IPI vs. DEL + NCCN-IPI: 0.645 vs. 0.674, *P* < 0.001. Similar to this study, their DEL + NCCN-IPI discrimination remained below an acceptable level (c-index ≥ 0.70) [22].

This confirmatory prognostic factor research of DE provided a rationale for the sample size, adhered to REMARK reporting guidelines, and performed IHC on whole tissues. Limitations in the partial availability of FISH for *MYC/BCL2/BCL6* rearrangements (28%) and PET-CT restaging (22%) may have affected the case recruitment and outcome assessment. The cohort of 111 patients was small and may not justify the c-index as a definite statistical explanation.

## Conclusions

Adding MYC/BCL2 double expression to the NCCN-IPI may not improve the prognostic value to an acceptable level in resource-limited settings. Multiple independent confirmatory studies from a large cohort of lymphoma registries, excluding DHL/THL, would provide more evidence for the clinical utility of DE.

### Supplementary Information

**Additional file 1: Table 1.** REMARK profile – double expression of MYC/BCL2 to National Comprehensive Cancer Network Internal Prognostic Index.

## Data Availability

The data presented in this study are available upon request from the corresponding author.
