# Supplementary material for: Adding MYC/BCL2 double expression to NCCN-IPI may not improve prognostic value to an acceptable level
Source: Blood Res. 2024 Feb 19;59(1):2. doi: 10.1007/s44313-024-00006-w (PMC10903517; doi:10.1007/s44313-024-00006-w)
Supplement: Supplementary file 1 — Additional file 1: Table 1. REMARK profile – double expression of MYC/BCL2 to National Comprehensive Cancer Network Internal Prognostic Index. [file 44313_2024_6_MOESM1_ESM.docx]

**Supplementary Materials**

**Table 1.** REMARK profile – double expression of MYC/BCL2 to National Comprehensive Cancer Network Internal Prognostic Index.

| a) Patients and variables | | | | | |
| --- | --- | --- | --- | --- | --- |
| Study and marker | **Remarks** | | | | |
| Marker | M=Double expression of MYC and BCL2 proteins  (DE: MYC ≥ 40% and BCL2 ≥ 50%, non-DE: other than DE) | | | | |
| Further variable | v=NCCN-IPI | | | | |
| Patients | **n** | **Remarks** | | | |
| Assessed for eligibility | 319 | **Disease**: diffuse large B-cell lymphoma  **Patient source**: Ramathibodi Hospital and Thammasat University Hospital from 1 January 2014 to 31 Jan 2018 | | | |
| Excluded | 208 | 133 no R-CHOP, 75 no MYC and BCL2 | | | |
| Included | 111 | Age ≥18 years old, R-CHOP, MYC/BCL2 result | | | |
| Outcome events | 51 | Progression: refractory disease or relapse or death from any cause | | | |
| b) Statistical analysis | | | | | |
| Analysis (A) | **n** | **Events** | **Outcomes** | **Variables considered** | **Results/remarks** |
| IDA: Descriptive | 111 | 51 | Progression | DE | Fig. 1, Table 2, Fig. 2 |
| A1: Univariable  Unadjusted  A2: Adjusted | 111  111  111  111 | 51  47  51  51 | PFS  5-y PFS  PFS  PFS | M, v1  M, v1  M  M, v1 | Fig. 3 (KM)  Table 3 (Probability)  Table 4 (HR)  Table 4 (Adjusted HR and c-index of NCCN-IPI with DE) |

A: analysis; c-index: concordance index; DE: double expression of MYC and BCL2; ECOG: Eastern Cooperative Oncology Group Performance Status; HR: hazard ratio; IDA: initial data analysis; KM: Kaplan–Meier survival curves; LDH: lactate dehydrogenase; M: marker; NCCN-IPI: National Comprehensive Cancer Network Internal Prognostic Index; PFS: progression-free survival; R-CHOP: rituximab, cyclophosphamide, doxorubicin, vincristine, and prednisolone; v: variable.
